# Practical Observations on Rheumatic Ophthalmia; with Cases

**Published:** 1827-01

**Authors:** William Mackenzie

**Affiliations:** Andersonian Professor of Anatomy and Surgery, and One of the Surgeons to the Glasgow Eye Infirmary.


### RHEUMATIC OPHTHALMIA.

*Practical Observations on Rheumatic Ophthalmia; with Cases.* By WILLIAM MACKENZIE, Andersonian Professor of Anatomy and Surgery, and one of the Surgeons to the GLASGOW EYE INFIRMARY.

IN a former paper,* I stated that the three inflammatory diseases of the eye, which most frequently arise in adults from atmospheric influences, were the *catarrhal*, the *rheumatic*, and the *catarrho-rheumatic*; and I gave some account of the first of these ophthalmiæ, and of the method of treatment.

I now proceed to the Rheumatic, of which the following particulars are sufficiently diagnostic.

1. *Seat of the disease.*—The catarrhal ophthalmia is an affection of the conjunctiva: the rheumatic has its seat in the albuginea, sclerotica, and periosteum within and round the orbit.

2. *Redness.*—The redness in the catarrhal is reticular, and the turgid vessels are evidently conjunctival: in the rheumatic, the chief redness is zonular, and seated under the conjunctiva.

3. *Nature of the inflammation.*—The catarrhal is an inflammation of a mucous membrane, and is a blenorrhœal or profluvial disease, attended with an increased and morbid secretion of mucus: the rheumatic attacks the fibrous membranes of the organ of vision, and is unattended by any morbid secretion.

4. *Pain.*—The pain in the catarrhal ophthalmia arises on the surface of the conjunctiva, is compared to the sensation of roughness, or to the feeling which might be excited by sand or broken glass under the eyelids; does not extend to the head; and is felt most in the morning, or when the eyes

* Number for October, page 317.

begin to be moved: the pain of the eyes in the rheumatic ophthalmia is pulsative and deep-seated; the chief pain, however, is not in the eye, but round the orbit, in the eye-brow, temple, cheek, and side of the nose, and is severely aggravated from sunset till morning.

*Degree of frequency.*—The pure rheumatic ophthalmia is comparatively a rare disease. For one case of pure rheumatic, we may meet with perhaps ten cases of catarrhal ophthalmia, and six of that mixed disease called catarrho-rheumatic, in which both conjunctiva and sclerotica are affected, and the symptoms of the two former ophthalmiæ combined.

*Exciting causes.*—Rheumatic ophthalmia may be distinctly traced, in most instances, to exposure of the eye to a continued blast of cold air, while the head and face are in a state of perspiration. The patient, in the history which he gives of his case, commonly mentions some particular exposure of this sort, soon after which the redness and rheumatic pain commenced: for example, sleeping with the head exposed to the air entering by a chink of the wall, or by a broken pane of glass; travelling during the night, with one side of the head close to the broken window of a carriage; suddenly issuing from a crowded room into the cold air of the street; exposure to the blast which flows from the stage into the body of a theatre; keeping wet clothes on the head when over-heated.

I have not observed that this disease is much more apt to occur at one season of the year than another. It is certainly much more apt to attack persons in middle life, than either the young or the old. Indeed, I have never seen it in children, nor in those far advanced in life. Probably the same exciting causes which, in persons of middle life and robust constitution, are apt to induce rheumatic ophthalmia, would in a child excite catarrhal or pustular ophthalmia, and in an old person the catarrho-rheumatic. Rheumatic ophthalmia is very apt to re-attack an individual who has already suffered from it on a previous occasion.

The mode of distribution of the inflamed vessels in rheumatic ophthalmia has been very correctly represented by Mr. Wardrop in the Medico-Chirurgical Transactions, Vol. X. Plate I. The fasciculi of vessels which advance in radii to the edge, and sometimes even a little over the edge of the cornea, are larger and more turgid than the radiating vessels seen in iritis, and seem more on the surface of the sclerotica.

In general, there is no tendency to chemosis in the pure rheumatic ophthalmia, nor do the eyelids take part in the disease.

Dimness of vision uniformly attends this disease, depending on an accompanying haziness of the cornea and pupil, attended by slight contraction of the pupil and sluggishness in the movements of the iris. If only one eye is affected, which is generally the case, (at least for some time,) the pupil of that eye is seen at once to be less than that of the sound eye. The iris becomes even slightly discoloured; it becomes greenish, for instance, if naturally blue; and the attending iritis may even go on to evident effusion of coagulable lymph within the pupil. It must be understood, however, that a severe degree of iritis rarely attends this rheumatic sclerotitis.

Except haziness of the cornea and of the pupil, which may be attributed to slight effusion, it has never happened to me to witness any other of the secondary phenomena of inflammation in pure rheumatic ophthalmia. I have not seen the disease terminate in any form of suppuration or of ulceration, both of which are very common in the catarrho-rheumatic ophthalmia.

The access of light does not in general prove very distressing to the patient in rheumatic ophthalmia. The affected eye feels dry and hot in the early period of the disease; but after a time, especially after the symptoms are somewhat abated by blood-letting, there is considerable epiphora.

The pain which attends the rheumatic ophthalmia at its commencement is of a stinging kind, and extends from the eyeball to the whole region of the orbit. It is strikingly augmented by warmth. It often affects the forehead, the cheek-bone, and the teeth; extending sometimes even to the lower jaw. Occasionally it is precisely confined to one half of the head. In some instances it is severe on the side, or even in the cavity, of the nose, or in the ear. But, above all, the eyebrow, cheek, and temple, are its chief seats.

The pain is not unfrequently the acute pulsatory pain of phlegmon; in other cases it consists rather in an agonising kind of feeling, which distresses and wearies out the patience of the person affected. It never ceases entirely, so long as the disease continues; but it varies much in degree, coming on with severity about four, six, or eight o'clock in the evening, continuing during the night, becoming most severe about midnight, and abating towards morning; till then totally preventing sleep, and occasioning great distress. The patient never fails, in the history he gives of his case, to insist on the nocturnal pain, and with his finger to describe and point out its circum-orbital seat. It is much more in the forehead, temple, cheek, and side of the nose, than in the eye.

Mr. Wardrop appears to be inclined to explain the peculiarity of the pain in this disease, from the sympathy which the sclerotica has with the adjacent structures of the same nature. Perhaps, we ought rather to conclude that the periosteum round the orbit, and the fascia of the temporal muscle, (similar structures to the sclerotica,) have become affected at the same time, in a similar way, and from the same exciting cause.

*Constitutional symptoms.*—Symptomatic fever accompanies this disease, increasing along with the nocturnal paroxysm of pain. The pulse becomes frequent, and sometimes strong, full, and hard. The tongue is white and furred, and the mouth ill-tasted; there is more or less nausea, and the skin is hot and dry. The digestive organs are deranged, the appetite impaired, the bowels confined, and the excretions morbid.

The progress and severity of the disease vary much in different cases. In some, the attack is very slight, and soon goes off, without permanently injuring the organ. At other times, it is extremely severe, continues long, and, if misunderstood, may ultimately destroy the eye. Not unfrequently, the disease falls into a chronic state, without being very severe.

The best description of this disease which I have met with, is unquestionably that by Mr. Wardrop; but as I have attempted to gather the symptoms from a minute examination of the cases which have fallen under my care, I have ventured to differ, both from Mr. Wardrop and from Professor Beer, in several particulars.

### TREATMENT.

1. *Blood-letting.*—In plethoric persons, with a full and hard pulse,—indeed almost always in rheumatic ophthalmia,—it is necessary to take away blood from the arm, and to apply leeches to the temple. I feel myself obliged entirely to differ from Mr. Wardrop, in his opinion that patients affected with rheumatic ophthalmia neither bear bleeding to a great extent, nor are alleviated by this remedy. Mr. Wardrop has even stated the little relief afforded by bleeding in this disease, as one of its diagnositic characters. This entirely disagrees with my experience, and is, I apprehend, altogether contrary to what we observe in other rheumatic affections. Bleeding, both general and local, I have uniformly found extremely useful in rheumatic ophthalmia, and

I believe it ought to be employed in almost every case. The first night after taking fifteen or twenty ounces of blood from the arm, the patient is generally so much relieved as to get some sleep, even though no other remedy be employed. Next day, I am in the habit of applying a dozen of leeches to the temple; but, if the pulse be still strong and full, and the circum-orbital pain not relieved, I first repeat the venesection. I regard blood-letting as of the most urgent necessity in this disease: in no species of ophthalmia is this remedy so necessary, or so remarkable in its good effects.

2. *Calomel and Opium.*—In rheumatic ophthalmia, I have never failed to find this combination highly useful, in checking the circum-orbital pain, restoring the digestive system to its healthy state, exciting the skin, and dissipating the redness of the eye. Two grains of calomel, with one of opium, are to be continued every evening till the gums begin to be affected, when the calomel may be omitted, and ten grains of Dover's powder substituted in place of the opium. Mr. Wardrop states that mercury, when given in this disease so as to produce ptyalism, aggravates more than mitigates the symptoms. This does not correspond with what I have observed. I do not, indeed, push the mercury in order to affect the mouth, but I have not witnessed any bad effects from the mouth becoming sore.

3. *Opiate Frictions.*—The patient experiences great relief from carefully rubbing the forehead around the orbit with warm laudanum. Beer used opium moistened with saliva. Friction with either of these substances assuages the pain, if already present; but ought rather to be employed about an hour before the nocturnal paroxysm is expected, which it will greatly lessen, and sometimes entirely prevent. In chronic cases, equal parts of laudanum and tincture of cantharides may be used for this purpose.

4. *Blisters,* repeatedly applied behind the ear, and to the temple, but above all a large blister to the nape of the neck, will be found useful.

5. *Vinum Opii.*—Applications to the eye itself have but little power over this disease. Those which are so useful in other ophthalmiæ, are often hurtful in the rheumatic. The lunar-caustic drops, for instance, which may be regarded as specific in catarrhal ophthalmia, are in the present disease decidedly injurious. When all the febrile and painful symptoms, however, are gone, and little more than lingering redness with weakness of the eye remains, the vinum opii in a diluted state will be found beneficial, dropped upon the eye twice or thrice, or the pure vinum opii once, a-day.

6. *Belladonna.*—During the whole course of rheumatic ophthalmia, the pupil of the affected eye ought to be kept under the influence of belladonna, either by smearing the moistened extract over the eyebrow and eyelids every evening at bedtime, or by infusing one drachm of the extract in each ounce of the laudanum which is used for rubbing the head.

7. *Purgatives.*—A laxative glyster every morning, or a small dose of Epsom salts, may be given to obviate the constipating effects of the opium.

8. *Sudorifics.*—The warm pediluvium at bedtime, with warm diluent drinks towards evening, operating along with the opium, will in general sufficiently fulfil this purpose. Mr. Wardrop recommends antimonial powder, and Beer employed guaiac, for exciting the skin in this disease.

9. *Tonics.*—Small doses of cinchona, or of the mineral acids, will be found advantageous in the chronic stage of the disease, and during convalescence. In old mistreated cases, Fowler's solution gives great relief, in doses of from eight to twelve drops thrice a-day.

The first, second, third, and sixth of the remedies now enumerated, are to be had recourse to in the first instance. I have never seen these remedies fail in any acute case, however severe. Nor have I seen any permanent sequelæ left, when the disease was treated with bleeding, calomel, and opium, opiate friction, blisters, vinum opii, and belladonna.

CASES.

Not to load your pages with unnecessary repetitions and details, (which I am afraid your readers will accuse me of, on a former occasion,) I shall content myself with selecting three cases of rheumatic ophthalmia from the journals of the Glasgow Eye Infirmary.

Case I.—June 10th, 1825; Isabella Gall, aged thirty-five. For the last twelve days, dimness of the sight of the left eye, redness of the conjunctiva, and particularly of the sclerotica; the vessels running in radii a little over the edge of the cornea. Severe pain in the eye, with hemicrania, much aggravated from twelve at night till five in the morning. Slight nebula of the cornea, and sluggishness in the motions of the iris. Attributes the complaint to exposure to cold.

Mitt. sanguis e brachio ad ℥ xij. vel. xv.—Hirudines viij. temp. sinistr. cras mane.—Belladonna ad palpebr. sinistr.—℞. Subm. Hydr. gr. v.; Pulv. Jalap. gr. xv. M. cap. q. p.

13th.—Pain of the head and of the eye much relieved, and the zonular redness less. An attack of pain from half-past four till six this morning.

℞. Subm. Hydr. gr. ij.; Opii gr. ss. M. fiant tales doses vj. cap. j. m. et v. —Vesicat. pone aurem sinistram.

15th.—An attack of pain this morning from six to seven.

Repetantur hirudines.

17th.—Symptoms greatly abated.

Cont[r] Pulvis Subm. Hydr. et Opii, m. et v.

22d.—Rather more pain in the eye.

℞. Subm. Hydr. gr. ij.; Opii gr. j. M. fiant tales doses vj. cap. j. m. et v.—Vesicat. temp. sinistro.

24th.—Instill. gtt. Vini Opii oculo sinistro.

29th.—Occasional attacks of pain deep in the eye.

Repetat. vesicatorium.—Cap. Pulv. Ipecacuan et Opii ℈j. horâ somni.

July 6th.—Dismissed cured.

Case II.—July 6th, 1825; John Martin, aged thirty. Severe ophthalmia rheumatica of the right eye, of eight days' standing. The redness of the conjunctiva and sclerotica is very considerable; the pain in the eye acute; cornea nebulous, and vision very dim. Severe pain in the night; pulse eighty-four, strong.

Venesectio ad ℥ xx.—℞. Subm. Hydr. gr. v.; Pulv. Jalap. gr. xv. M. cap. statim.

7th.—Symptoms not abated.

Repetatur venesectio.—Hirudines xij. temp. dextr.—Sulph. Magnesiæ ℥ ij. —Perfric[r] regio frontalis Tr. Opii bis indies.

8th.—Pain of the eye somewhat relieved.

Vesicat. magnum ad nucham, et postea Unguent. Pulv. Cantharidis.—H. s. Pulv. Ipecac. et Opii ℈j.

10th.—Eye and temple much easier since twelve o'clock last night. Redness not diminished, and dimness of the cornea increased. No stool.

Repetatur Pulvis purgans statim.—Belladonna palp. dextr.—Cont. Pulv. Ipecac. et Opii h. s.

11th.—Redness a little diminished; cornea clearer, and vision not so dim. Eye continues easier, but there is more epiphora. Perspires freely through the night. Three stools from the powder and some salts which he took this morning.

Contin[r] Belladonna et Pulv. Ipecac. et Opii.

13th.—Symptoms much abated.

Hab. Pil. Colocynth. comp. ij. o. n.—Cont. Pulv. Doveri.

20th.—Instill. gtt. Vini Opii oculo dextro.

29th.—Symptoms gone, except weakness of the eye.

Case III.—December 30th, 1825; Mary Scott, aged twenty. Rheumatic ophthalmia of three weeks' standing, particularly affecting the left eye, the cornea of which is muddy and vision dim. Severe pulsating pain in the eyes, and severe pain in the supraorbital and temporal regions, from twelve P.M. till seven A.M. Eyes irritable, and they water much. Pupils sluggish in their motions, and the sight irregular. Was affected with a bowel complaint, subsequent to jaundice, at the time when her eyes became affected. Has applied leeches to the eyelids and temples, without relief. Feels chilly through the night.

Venesectio ad ℥ xv. vel. xx.—Cras mane Hirudines vj. temp. singulis.—℞. Subm. Hydrarg. gr. ij.; Opii gr. j. M. fiant tales doses vj. capiat j. omni nocte.—Fricetur pars capitis dolens Tr. Opii.—Pediluvium tepidum h. s.

January 2d, 1826.—Pain of the eyes, forehead, and temples much relieved by the venesection, so that the leeches were not applied. Zonular redness of both scleroticæ still considerable, but corneæ not so dim, and vision clearer. Perspired in the night. No noctural pain last night, which she attributes to the rubbing with Tr. Opii.

Cont. pulvis, fricatio, et pediluvium.

4th.—Redness much diminished; corneæ clearer, and vision perfectly distinct.

Cont. medicamenta.

6th.—Symptoms almost entirely gone.

Cont. medicamenta.

9th.*—Redness of the left conjunctiva; left lower eyelid swollen and red.

Instill. gtt. Sol. Nitr. Argent. oculo sinistro.

11th.—Right eye perfectly well. Still a little redness of the left.

Instill. gtt. Vini Opii.

16th.—Catarrhal discharge from the left eye.

Instill. gtt. Sol. Nitr. Argent.—Collyr. Mur. Hydr.—Ung. Præ. Rub. o. n.

18th.—Redness of the left eye less, and catarrhal discharge ceased.

27th.—Dismissed cured.

* In the interval between the 6th and the 9th, this patient, cured of the rheumatic ophthalmia of both eyes, in consequence probably of some new exposure, caught catarrhal ophthalmia of the left eye. This case differs from those which I shall hereafter describe under the name of Catarro-rheumatic, in this respect, that here the catarrhal inflammation succeeded the rheumatic; whereas, in catarrho-rheumatic cases, the conjunctiva and sclerotica are affected simultaneously.

